# The 20th International Conference on Radionuclide Therapy (ICRT 2025)

**DOI:** 10.1055/s-0045-1814142

**Published:** 2026-01-06

**Authors:** Savvas Frangos

**Affiliations:** 1 Department of Nuclear Medicine, Nuclear Medicine and Thyroid Cancer Clinic, Bank of Cyprus Oncology Center, Nicosia, Cyprus

From November 6–9, 2025, the 20th International Conference on Radionuclide Therapy (ICRT 2025) was organized in Limassol, Cyprus, with the motto “Back to the Roots” because it returned to the place where it was born.

Cyprus has been connected to both the *World Journal of Nuclear Medicine (WJNM)* and the International Conference on Radionuclide Therapy (ICRT) since their creation.

The connection with the *WJNM* dates back to its founding in 2002. The journal was edited by Ajit Padhy, produced by me, and printed here on the island. The first issue, Volume 1, Number 1, came out in October 2002. Today, we are at Volume 24, Number 3. The *WJNM* will publish in this issue the abstracts presented at ICRT 2025.

**Fig. 1:**
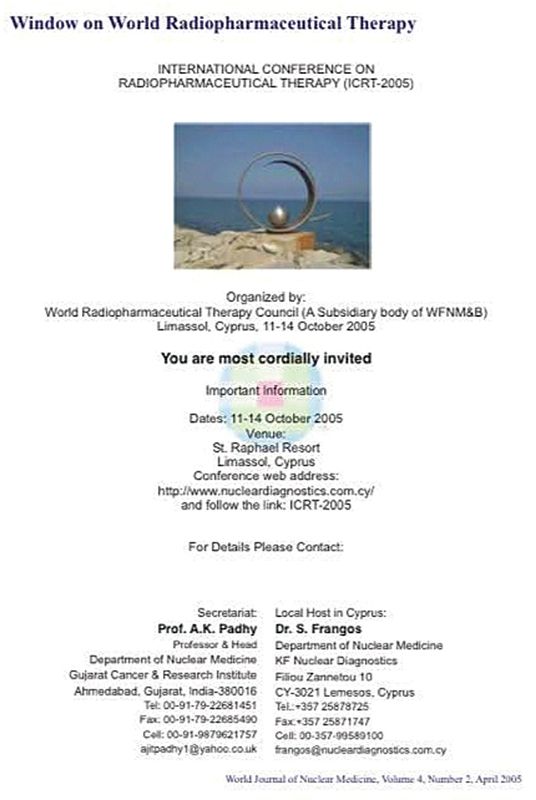
The announcement of the first ICRT in Cyprus in 2005.

The connection with ICRT goes back to the very first idea of organizing a dedicated conference on Theragnostics. It was in 2005 when I received an email from the late Ajit Padhy, who had just left the IAEA, inviting me to organize an international conference under the name International Conference on Radionuclide Therapy. There was no budget and no available funding, but we successfully organized the first conference from October 11–14, 2005. This was before the establishment of the World Association of Radiopharmaceutical and Molecular Therapy (WARMTH), and the conference was organized by the World Radiopharmaceutical Council. Since then, it has become a tradition. The first announcement regarding the conference was published in Volume 5, Number 2 of *WJNM* ( Fig. 1 ).

**Fig. 2:**
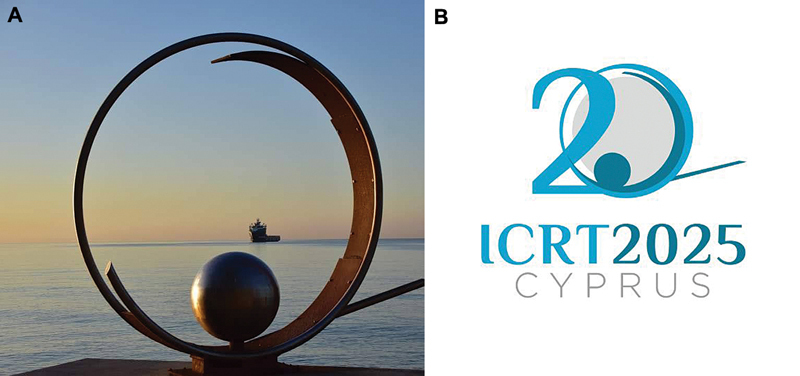
The “Frozen Wave” sculpture and the ICRT 2025 logo.

A long-standing tradition of the conference is the use of a local sculpture as its symbol. Continuing this tradition, we used, 20 years later, the same symbol as in 2005: the “Frozen Wave” located on the Limassol Promenade. The sculpture was incorporated into the ICRT 2025 logo ( Fig. 2 ).

**Fig. 3:**
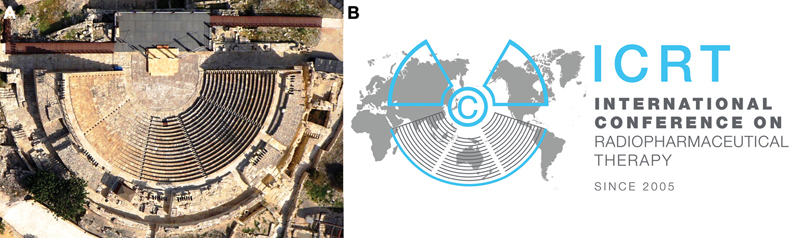
The Curium Amphitheater and the ICRT logo.

In addition, the general ICRT logo was inspired by the Curium Amphitheater, located west of Limassol ( Fig. 3 ).

At this year's International Conference on Radionuclide Therapy, we celebrate 20 years ICRT. We hosted delegates from 29 countries around the globe, with all continents represented. The faculty included experts from 14 countries. A total of 24 outstanding posters were presented during the two poster walks.

We had two prestigious awards: the Best Poster Award, sponsored by the World Federation of Nuclear Medicine and Biology, and the Oncidium Poster Award, sponsored by the Oncidium Foundation.

The Best Poster Award was given to Sugandha Dureja for the work titled “Response-Adapted PSMA Radioligand Therapy Without Dosimetry: A Feasible Model for Low-Resource Settings” authored by Sugandha Dureja, Anjali Gupta, Tarsem Singh, and Vinay Kumar.

**Fig. 4:**
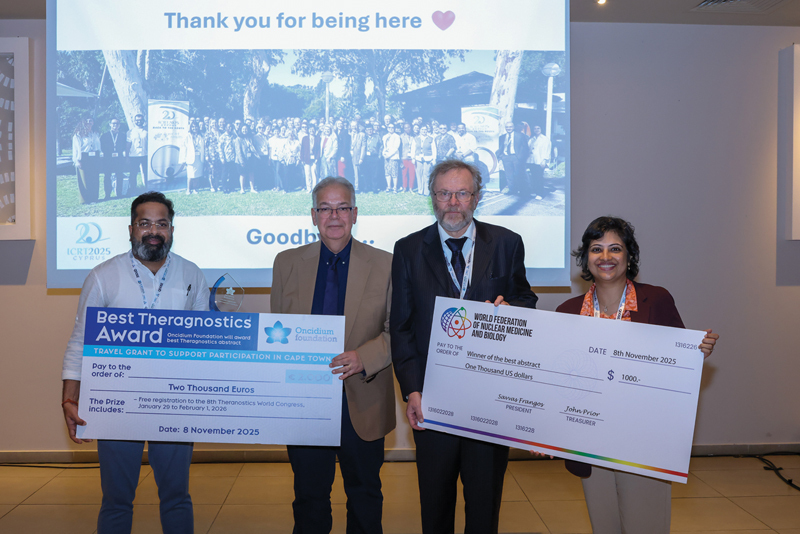
The winners of the best abstracts with the conference organizers. Swagat Dash (Oncidium award), Savvas Frangos (ICRT 2025 chair), Kalevi Kairemo (WARMTH president), and Sugandha Dureja (WFNMB award).

The Oncidium Award was given to Swagat Dash for the work titled “Feasibility and Safety of 161Tb-FAP2268 Peptide Targeted Radionuclide Therapy in Solid Tumors: Preliminary Clinical Insight from India,” authored by Swagat Dash, Vindhya Malasani, Kamal Yadav, Rakhee Vatsa, Priya Ashok, Parul Thakral, Majid Assadi, Habibollah Dadgar, Hossein Arabi, Ashok Kumar, Naveen Sanchety, Abhishek Raj, Vishnu Hari, and Dinesh Pendharkar ( Fig. 4 ).

The conference featured a special session dedicated to students, titled “From Molecules to Miracles: A Student Introduction to Nuclear Medicine,” with a large number of participants. The students showed extraordinary interest in Nuclear Medicine.

**Fig. 5:**
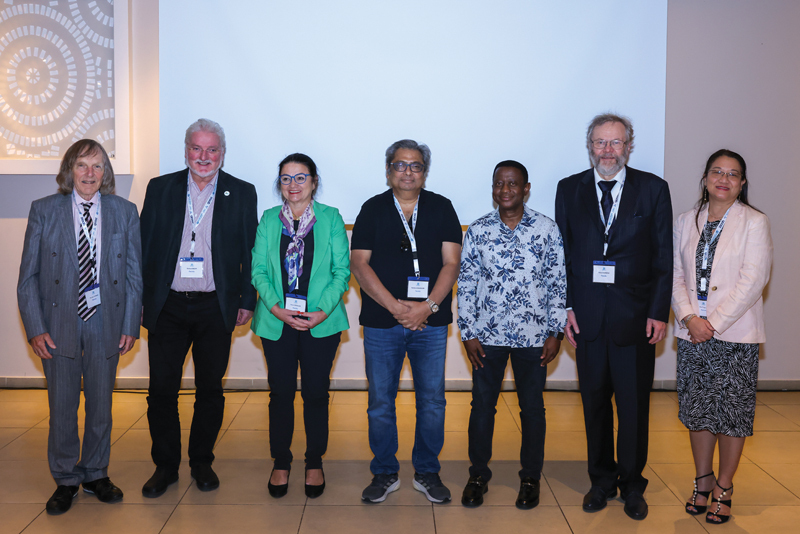
Past, current, and future Presidents of WARMTH: Harvey Turner, Richard Baum, Irene Virgolini, Partha Choudhury, Mike Sathekge, Kalevi Kairemo, and Sze Ting Lee.

We had the privilege of welcoming nearly all the Presidents of WARMTH. Only Suresh Srivastava was unable to attend. Of course, the absence of Ajit Padhy was deeply felt ( Fig. 5 ).

It was a privilege and an honor to host, after 20 years, the International Conference on Radionuclide Therapy in the place where it originated. A special thank you to all participants, faculty members, and the organizing committee for their efforts.

Savvas Frangos, FEBNM

Chair, ICRT 2025

